# Case Report: Overcoming challenges in pancreatic cancer with liver metastases: a personalized therapeutic odyssey of TACE, ablation, and immunotherapy

**DOI:** 10.3389/fimmu.2023.1275782

**Published:** 2023-10-11

**Authors:** Ying Zhu, Zhouyu Ning, Zhiqiang Meng

**Affiliations:** ^1^ Department of Integrative Oncology, Fudan University Shanghai Cancer Center, Shanghai, China; ^2^ Department of Oncology, Shanghai Medical College, Fudan University, Shanghai, China

**Keywords:** pancreatic cancer, immunotherapy therapy, transarterial chemoembolization (TACE), PD-L1, PD-1, microwave ablation

## Abstract

Pancreatic cancer represents a malignant neoplasm originating from pancreatic cells. The optimal approach to cancer treatment remains uncertain, lacking a definitive consensus. Here, we present a compelling case of a 49-year-old female with pancreatic head cancer with liver metastases, as identified by CT and confirmed by biopsy. PET-CT indicated widespread metastatic involvement. TACE therapy with gemcitabine and cisplatin was initiated, yielding a stable disease response. The patient’s high PD-L1 expression prompted TACE-PD-1 monoclonal antibody combination therapy. Subsequent treatments, including ablation, sustained PD-1 immunotherapy, and consolidation TACE, culminated in a complete response, as evidenced by imaging and tumor marker dynamics. Our case underscores the potential of multifaceted strategies in managing aggressive pancreatic cancer.

## Introduction

Pancreatic cancer (PC) is a formidable malignancy with a mere 11% 5-year survival rate (1). While strides in immunotherapy and targeted interventions have propelled favorable outcomes across various malignancies (2, 3), these strides have yet to encompass PC. Surgical intervention emerges as the primary treatment avenue for localized early-stage pancreatic cancer, whereas chemotherapy prevails as the frontline regimen for advanced and metastatic cases (4–6). Although surgical intervention thrives in managing early-stage cases, most patients present with advanced stages, often accompanied by liver metastases, rendering them ineligible for surgical intervention. Thus, the quest for pioneering and efficacious therapeutic avenues paramount significance in elevating survival rates and enhancing the quality of life for pancreatic cancer patients.

Transarterial chemoembolization (TACE), a minimally invasive interventional radiology technique orchestrating the targeted delivery of chemotherapy and embolic agents via the arterial blood supply, has been integrated with systemic chemotherapy in hepatocellular carcinoma management, yielding significant survival extensions (6, 7). Though the application of TACE in addressing pancreatic malignancies encounters hurdles due to the anatomical intricacies and distinctive biological behavior of pancreatic cancer (8), the potential efficacy of TACE alongside systemic chemotherapy shows a promising therapeutic course for those individuals with concurrent hepatic metastases. TACE delivers concentrated chemotherapy agents directly to intrahepatic metastases, facilitating localized control of tumor proliferation. Furthermore, embolic agents exert a pivotal role in interrupting tumor blood supply, ameliorating the tumor microenvironment, and potentiating the cytotoxic impact of chemotherapeutic agents. Cooperatively, the adjunctive use of systemic therapy (including chemotherapy, targeted modalities, immunotherapy, etc.) functions systemically to inhibit tumor propagation and progression. Microwave ablation harnesses microwave energy to heat tumor cells rapidly, expediting the generation of high temperatures and making it particularly suited for more extensive and challenging-to-access tumor lesions (9). Its application in liver cancer therapy is attracting increasing interest (10). Moreover, within the realm of clinical practice involving pancreatic cancer liver metastases, microwave ablation emerges as a promising innovative approach that augments the overall therapeutic efficacy by diminishing tumor foci (11). Concurrently, despite PD-1 monoclonal antibody therapy not yet attaining the status of a definitive standard for pancreatic cancer treatment, its potential therapeutic gains in patients exhibiting elevated PD-L1 expression warrant careful consideration (12).

Consequently, combining diverse treatment modalities such as TACE, microwave ablation, and immunotherapy unfolds expansive vistas for creating custom therapeutic regimens for individual patients. This comprehensive and multimodal treatment strategy holds the potential to yield marked clinical advancements and survival benefits for individuals navigating the complexities of pancreatic cancer accompanied by liver metastasis. Here, we present a compelling case study of a PC diagnosis achieved through CT imaging and liver biopsy. Our patient underwent a comprehensive therapeutic approach, synergizing TACE, microwave ablation, and immunotherapy, yielding satisfying therapeutic outcomes with commendable tolerability. The amalgamation of TACE, microwave ablation, and immunotherapy effectively enhances the therapeutic impact in managing PC, surpassing the confines of singular modalities.

## Case presentation

A 49-year-old female was found to have a pancreatic head mass and multiple low-density lesions in the liver on computed tomography (CT) in October 2020, consistent with pancreatic head cancer with liver metastases. A liver biopsy was conducted, and subsequent cytological analysis confirmed the presence of cancer. Positron emission tomography-computed tomography (PET-CT) showed a pancreatic mass with high 18F-fluorodeoxyglucose (FDG) uptake, suggesting metastasis, liver metastasis, portal vein tumor thrombus, and metastasis to peripancreatic and retroperitoneal lymph nodes. Histopathological examination of the liver biopsy showed poorly differentiated carcinoma with an adenocarcinoma-like tendency. Based on the clinical presentation, pancreatic cancer with metastasis cannot be excluded. Notably, there exists an absence of prior tobacco and alcohol consumption within the patient’s history, as well as an absence of previous exposure to pharmaceutical treatments. There is no reported history of malignant tumors within the patient’s familial lineage.

Subsequently, the patient initiated a treatment plan involving TACE (transarterial chemoembolization) therapy, which coincided with gemcitabine, cisplatin, and pirarubicin (THP). These sessions occurred on October 28, November 24, and December 18, 2020. The procedure encompassed the administration of gemcitabine at a dose of 0.8mg and cisplatin at 30mg into the celiac trunk and superior mesenteric artery, correspondingly. Additionally, a dosage of THP60mg and super liquefied lipiodol at 2ml was introduced into the liver tumor’s feeding artery via the superior mesenteric artery route.

On December 18, a CT scan revealed infiltration of the portal vein and superior mesenteric vein by the pancreatic neck mass, with suspected involvement of celiac trunk branches. Multiple metastatic lymph nodes were also observed in the peripancreatic region, retroperitoneum, and porta hepatis. The liver exhibited various metastatic nodules, some showing changes after treatment. Relative to the CT scan findings from October 14, 2020, the tumor size was not significantly reduced and didn’t escalate in its dimensions; hence, the patient’s response to the three times TACE interventions was evaluated as stable disease (SD).

**Figure 1:**
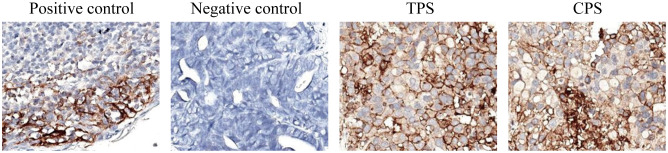
The PD-L1 test results from the patient’s tumor biopsy sample. The TPS is over 95%, and the CPS is over 90. (TPS, tumor proportion score, the ratio of PD-L1 stained tumor cells to total tumor cells x 100%; CPS, combined positive score; the ratio of PD-L1 stained cells to total tumor cells, including tumor cells, lymphocytes, and macrophages x 100. The negative expression of PD-L1 is TPS < 1%; the low expression is TPS > 1 and < 49%; and the high expression is TPS ≥ 50%).

**Figure 2:**
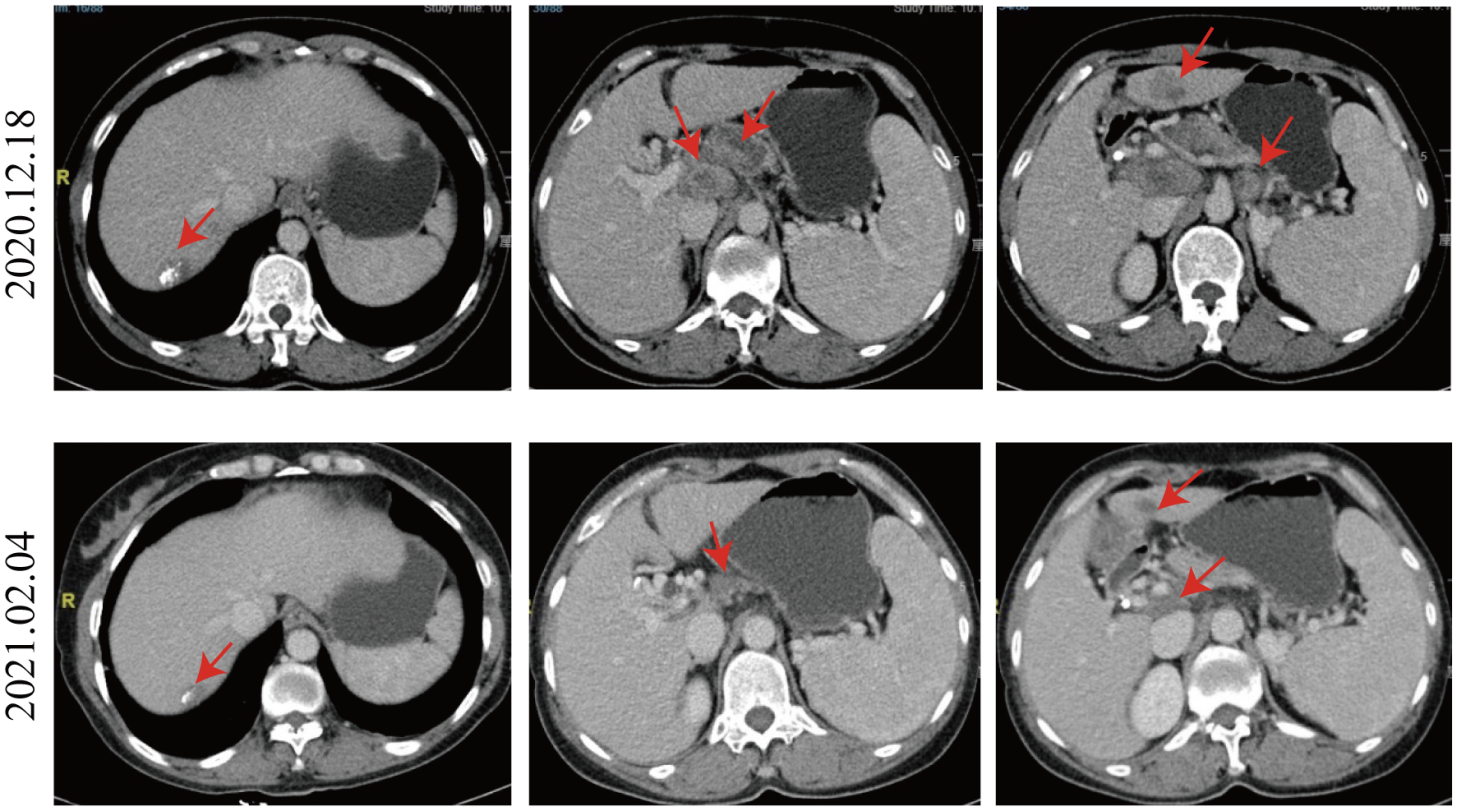
Comparison of CT images taken on December 18, 2020, and February 4, 2021. Compared to the CT image of 2020.12.18, the nodular masses of the pancreas’s neck, the liver’s hilar region, and the retroperitoneum were smaller. Multiple nodules were still present in the liver, and some exhibited slight changes following treatment, not significantly different from before.

Given the positive PD-L1 test results from the patient’s tumor biopsy sample (**Figure 1**), we adjusted the patient’s treatment strategy to TACE combine with a PD-1 monoclonal antibody therapy. Briefly, starting from December 19, 2020, the initiation of PD-1 inhibitor therapy has been established on a regimen of administration once every three weeks, devoid of interruptions. TACE interventions were conducted on January 13, February 4, and March 11, 2021. The procedure involved injecting 1.6g of gemcitabine into the celiac trunk and superior mesenteric artery. Additionally, 200mg of albumin-bound paclitaxel (Abraxane) was administered into the hepatic tumor-feeding artery, while THP 60mg, along with 2 ml of highly liquid lipiodol, was introduced into the superior mesenteric artery’s feeding artery. In the follow-up CT on February 4, 2021, several smaller nodular masses were observed above the pancreas’s neck, the liver’s hilar region, and the retroperitoneum compared to previous scans. While multiple nodules were still present in the liver, some exhibited slight changes following treatment, although not significantly different from before (**Figure 2**). As a result, the collective impact of the three interventions, along with PD-1 immunotherapy, was classified as a partial response (PR).

**Figure 3:**
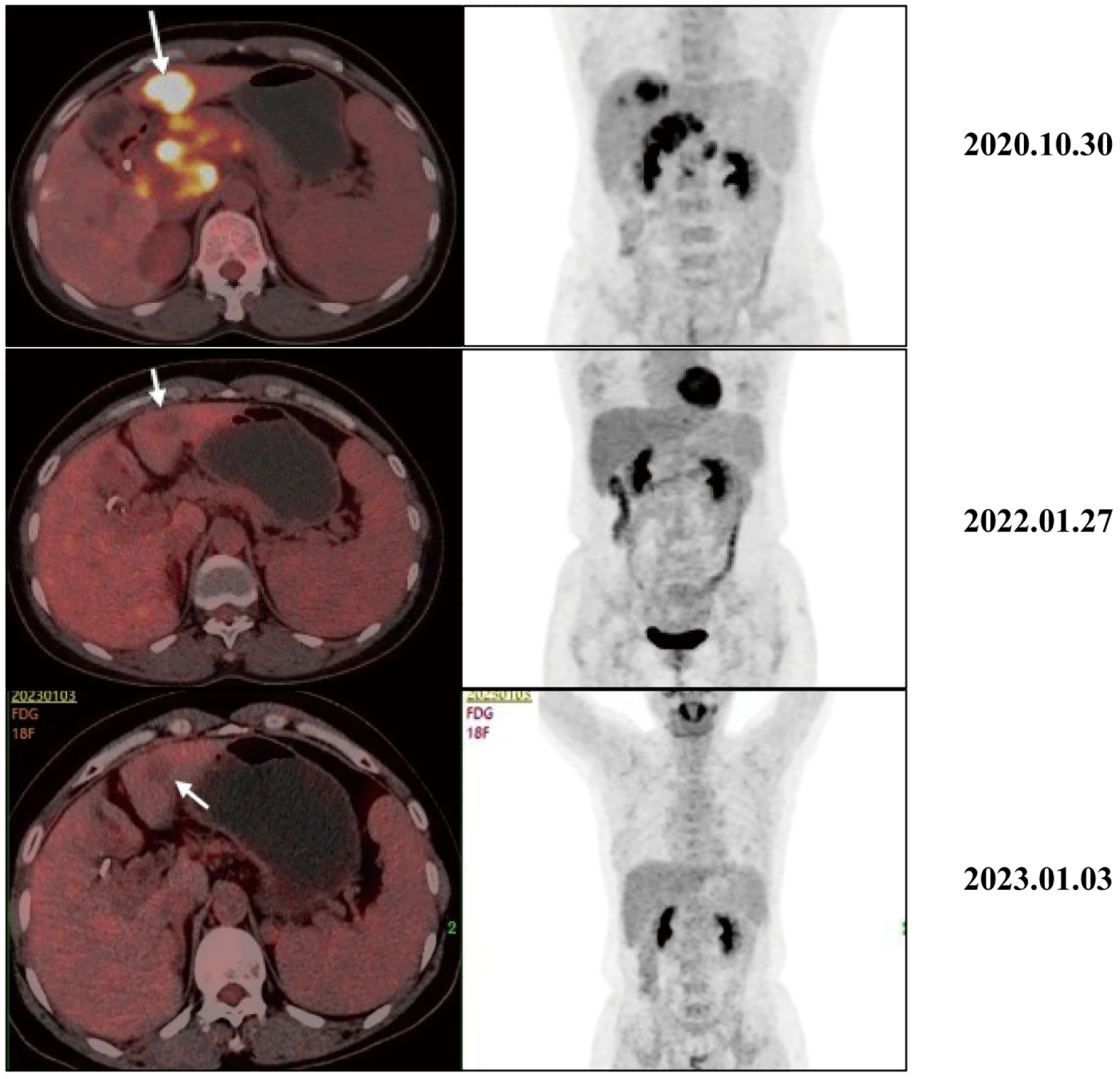
Typical PET-CT images at different time points before and after receiving treatment. The PET-CT of 2020.10.30 showed a mass with high FDG uptake, suggesting metastasis, liver metastasis, portal vein tumor thrombus, and metastasis to peripancreatic and retroperitoneal lymph nodes. After receiving treatment, intrahepatic lesions initially showed a significant decrease in size and FDG metabolism. In the 2022.01.27 and 2023.01.03 images, no increase in FDG metabolism was observed, and the primary peripancreatic and retroperitoneal metastatic lymph nodes and portal vein cancer thrombi have subsided.

**Figure 4:**
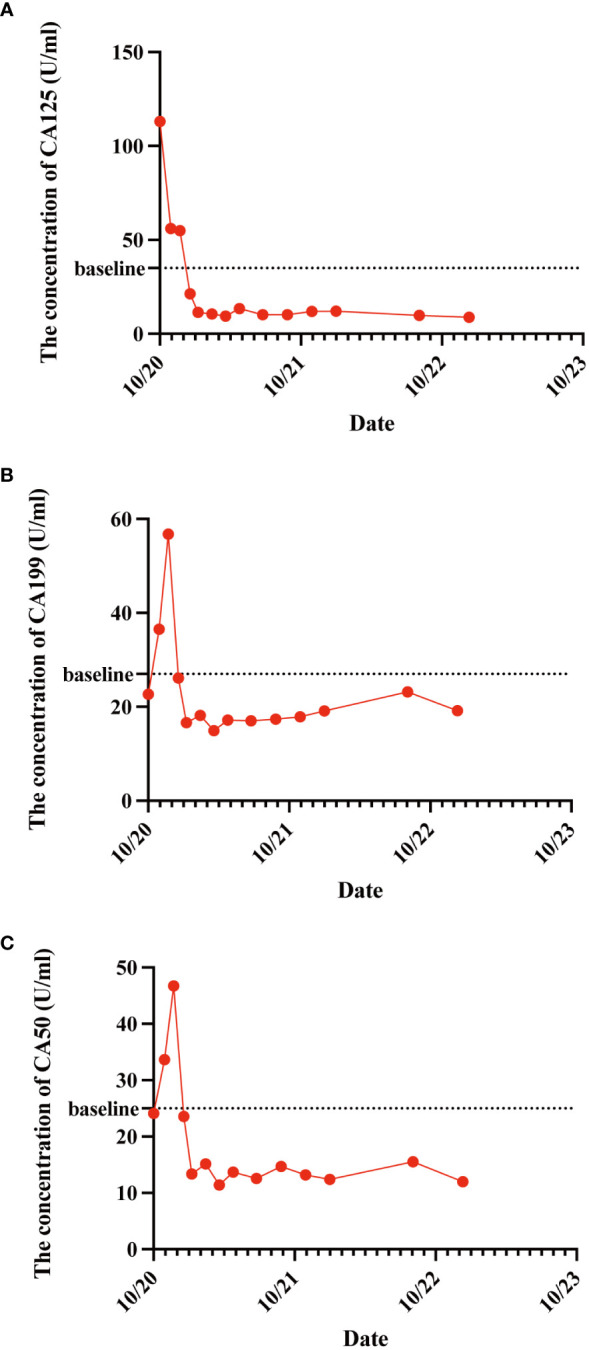
The variations in patient CA125 **(A)**, CA199 **(B)**, and CA50 **(C)** concentration levels before and after treatment administration. These tumor markers exhibited a transient elevation during the initial treatment phase and a subsequent decline below baseline levels.

As the reduced size of the liver metastatic lesions, in alignment with microwave ablation treatment protocols, microwave ablation of the liver metastasis was executed successfully on April 15, 2021, at our institution. The patient exhibited favorable postoperative recuperation. A subsequent magnetic resonance imaging (MRI) scan on May 20, 2021, revealed multiple nodules above the pancreas’s neck, within the hilar region of the liver and retroperitoneum, consistent with previous observations; Multiple liver masses with potential for post-treatment changes; Multiple small cysts in the liver may also be seen, with some edges slightly blurry. The strategy entails sustaining PD-1 immunotherapy to consolidate the therapeutic approach further. The administration regimen for PD-1 treatment involves a frequency of once every three weeks, with a dosage of 200 mg per session by intravenous infusion for one hour per session. Meanwhile, patients underwent TACE treatment on May 21, July 20, September 22, and November 24, 2021. Upon MRI evaluation on May 20, 2021, the tumor lacked developmental progression, primarily characterized by a perfusion-oriented nature, negating the necessity for embolization. Hence, the regiment deducted the THP 60mg and 2ml of highly liquid lipiodol, only injecting 1.6mg of gemcitabine into the celiac trunk and superior mesenteric artery and infusing 200mg of Abraxane into the abdominal trunk and superior mesenteric artery. Following these four consolidation TACE sessions, the patient’s therapeutic response consistently maintained a PR. Then, continue to PD-1 monoclonal antibody treatment consistent with the previous regimen. 2022.01.27 follow-up PET-CT showed no obvious space-occupying lesions, no increased FDG metabolism, two slightly low-density foci in the liver, and no increase in FDG metabolism. Peripancreatic and retroperitoneal metastatic lymph nodes, as well as the portal vein tumor thrombus, disappeared. These findings collectively indicated that the patients’ treatment had achieved complete response (CR). After that, the patients were treated with PD-1 monoclonal antibody consistent with the previous regimen, and PET-CT was examined again on 2023.01.03. The results were the same as those of 2022.01.27, indicating that the patients maintained the state of CR after PD-1 monotherapy (**Figure 3**). Furthermore, we supervised alterations in blood tumor markers, including CA125, CA199, and CA50, from the start of treatment (**Figure 4**), which exhibited a transient elevation during the initial treatment phase and a subsequent decline below baseline levels. CA199, the most prevalent and well-studied pancreatic cancer marker, measures tumor burden but not metastatic potential (13). CA 50, a tumor marker for pancreatic cancer, is typically used with CA 199 to assess the prognosis (14). CA125 is a tumor marker for ovarian cancer and the most significant predictor of pancreatic cancer with metastases in recent years (15, 16). Our clinical experience and prior studies show that monitoring CA199, CA125, and CA50 as a group can better assess the prognosis of pancreatic with metastases (15, 17). This observation underscores the effectiveness of TACE coupled with systemic therapy in this patient.

## Discussion

**Figure 5:**
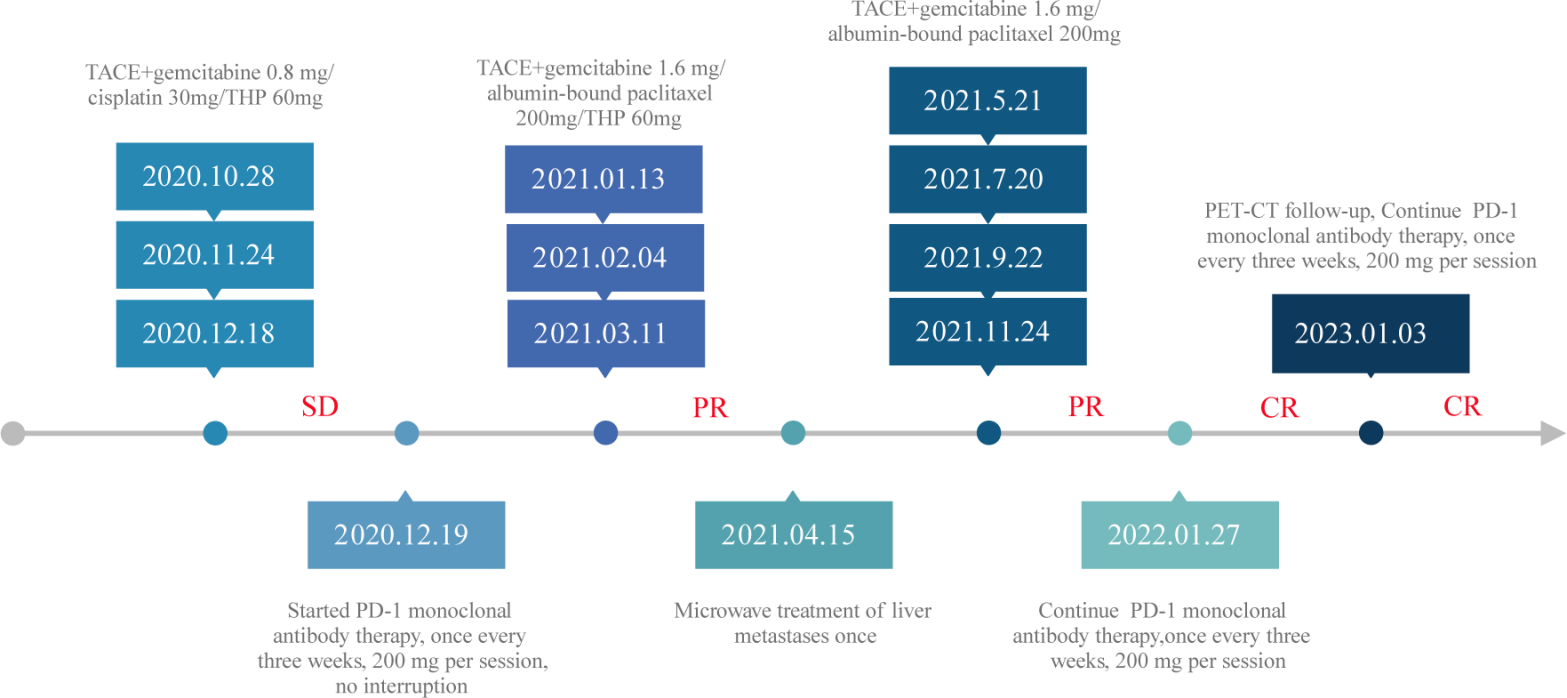
The timeline of the patient’s treatment.

This case report encapsulates a compelling therapeutic exploration and intervention journey in a 49-year-old female patient diagnosed with pancreatic head cancer and concurrent liver metastases. The timeline of the patient’s treatment is described in **Figure 5**. The complex coordination of precise therapeutic interventions, informed by advancing diagnostic knowledge, has significant ramifications for managing advanced cancerous conditions. The initial diagnostic process, driven by computed tomography (CT) and cytological investigation, revealed an intricate scenario marked by a tumor in the pancreas head and liver lesions that suggest metastasis. Significantly, the subsequent PET-CT scan revealed a range of metastatic spread extending from the pancreatic mass to the surrounding peripancreatic and retroperitoneal lymph nodes and a tumor thrombus in the portal vein. The histological examination of the liver biopsy provided further insight into the malignancy, revealing a poorly differentiated carcinoma with adenocarcinoma-like features, thereby reinforcing the ominous nature of the disease.

The treatment trajectory commenced with TACE interventions strategically synchronized with gemcitabine and cisplatin administration. These sessions yielded an SD status and paved the path for further refinements in the therapeutic strategy. The revelation of positive PD-L1 evaluation results from the patient’s tumor biopsy sample prompted a paradigm shift. A harmonized approach was adopted, combining TACE PD-1 monoclonal antibody therapy. The ensuing PD-1 immunotherapy regimen, marked by consistent administration, reflected the commitment to harnessing the potential of immunomodulation. Microwave ablation’s judicious application further accentuated the comprehensive approach. Its success in diminishing liver metastatic lesions demonstrated the merit of multidisciplinary interventions. Subsequent assessments, compared with earlier imaging, underscored the effectiveness of this strategy, revealing a transition to PR and emphasizing the cumulative effect of the therapeutic synergism. Continued vigilance and adaptability remained the cornerstone of this intricate endeavor. The strategic sequencing of subsequent TACE sessions fortified the therapeutic gains, culminating in a sustained PR. Notably, with the continuous treatment of PD-1 monoclonal antibody, the patient’s trajectory culminated in CR status, substantiated by the vanishing of metastatic foci, nodules, and tumor thrombus, as evidenced in imaging studies by follow-up PET-CT scans and blood tumor markers. The fluctuation of blood tumor markers, CA125, CA199, and CA50, presented a dynamic narrative, mirroring the treatment trajectory. The transient elevation during initial treatment, followed by a decline, underscored the intricate interplay between therapies and disease dynamics. This is a testament to the clinical relevance of tumor marker surveillance, reflective of therapeutic efficacy.

However, it is imperative to acknowledge that our case report has limitations. The study’s reliance on a solitary case report curtails how much our findings can be extrapolated to a broader populace. Furthermore, including a solitary patient in the study might not comprehensively encapsulate the diverse spectrum of individuals grappling with the intricacies of pancreatic cancer and liver metastases. Nevertheless, it is essential to underscore that our case represents an incipient stride towards tailoring treatment strategies in accordance with individual patient profiles. This pivotal endeavor serves as a bedrock for the precision-based management of malignancies. In the trajectory ahead, we strongly advocate for the inclusion of a broader and more appropriate cohort of patients to conduct methodologically robust clinical trials. This prospective undertaking holds promise in unearthing novel avenues for treating advanced pancreatic cancer, enriching the therapeutic landscape.

In conclusion, a significant factor contributing to CR in this case was our keen understanding of the patient’s disease trajectory, which enabled us to tailor treatment plans accordingly. Initially, a combination of TACE and chemotherapy was swiftly employed to stabilize the patient’s condition. After positive outcomes from the patient’s PD-L1 testing and considering their overall health, an opportunity for immunotherapy emerged. Promptly, PD-1 monoclonal antibody treatment was administered. Concurrently, we maintained vigilant surveillance of treatment response and strategically introduced TACE and microwave ablation during immunotherapy to enhance the release of tumor antigens, thereby amplifying the immune response. Consequently, this success can be attributed to the meticulous fusion of TACE, PD-1 monoclonal antibody therapy, and microwave ablation, harmonized with the patient’s individual medical profile. It is imperative to note that this innovative approach is an initial endeavor, and its replicability in yielding such favorable outcomes for other patients warrants further in-depth investigation through additional case studies.

## Conclusion

In summary, this case report underscores the remarkable efficacy of our combined therapeutic approach, showcasing the transformative potential of precision medicine in achieving and maintaining complete remission in advanced metastatic pancreatic cancer. Integrating TACE and PD-1 immunotherapy and ablative interventions, guided by comprehensive imaging and tumor marker monitoring, offers a promising paradigm for personalized treatment strategies in similar clinical scenarios. Our findings emphasize the importance of multidisciplinary collaboration and vigilant follow-up in optimizing patient outcomes. Further research and validation are warranted to solidify this innovative approach as a cornerstone in managing aggressive malignancies.

## Data availability statement

The original contributions presented in the study are included in the article/supplementary material. Further inquiries can be directed to the corresponding author.

## Ethics statement

The studies involving humans were approved by Fudan University Shanghai Cancer Center Institutional Review Board. The studies were conducted in accordance with the local legislation and institutional requirements. The participants provided their written informed consent to participate in this study. Written informed consent was obtained from the individual(s) for the publication of any potentially identifiable images or data included in this article. Written informed consent was obtained from the participant/patient(s) for the publication of this case report.

## Author contributions

ZM: Validation, Writing – review & editing, Funding acquisition, Resources, Supervision, Visualization. YZ: Conceptualization, Project administration, Data curation, Formal Analysis, Investigation, Methodology, Software, Validation, Writing – original draft, Writing – review & editing. ZN: Methodology, Resources, Supervision, Validation, Writing – review & editing.
